# Supplementary material for: Empirical methods for controlling false positives and estimating confidence in ChIP-Seq peaks
Source: BMC Bioinformatics. 2008 Dec 5;9:523. doi: 10.1186/1471-2105-9-523 (PMC2628906; doi:10.1186/1471-2105-9-523)
Supplement: Additional File 1 — A variety of html documents from the USeq web site detailing the available applications, their best usage, output file type descriptions, command line menus, etc. [file 1471-2105-9-523-S1.zip › USeqUserGuides/JavaDoc.zip!/JavaDoc/util/bio/annotation/ScoredCoordinate.html]

ScoredCoordinate


|  |  |  |
| --- | --- | --- |
| **Overview** \| **Package** \| **Class** \| **Use** \| **Tree** \| **Deprecated** \| **Index** \| **Help** | |  |
| **PREV CLASS**   **NEXT CLASS** | **FRAMES**    **NO FRAMES**     **All Classes** |  |
| SUMMARY: NESTED \| FIELD \| CONSTR \| METHOD | DETAIL: FIELD \| CONSTR \| METHOD |  |


---


## util.bio.annotation Class ScoredCoordinate

```
java.lang.Object
  util.bio.annotation.Coordinate
      util.bio.annotation.ScoredCoordinate
```

**All Implemented Interfaces:**: java.lang.Comparable

---

``` public class ScoredCoordinate extends Coordinate ```

---

**Constructor Summary**

`ScoredCoordinate(java.lang.String chromosome,
int start,
int stop,
double score)`


| **Method Summary** | |
| --- | --- |
| `double` | `getScore()` |
| `static ScoredCoordinate[]` | `makeRanked(Coordinate[] c)`             Returns an array of ScoredCoordinate where the score is the index position of the Coordinate[]. |
| `void` | `setScore(double score)` |
| `static java.util.HashMap` | `splitByChromosome(ScoredCoordinate[] sortedCoordinates)`             Split by chromosome into a HashMap of chromosome:ScoredCoordinate[]. |

**Methods inherited from class util.bio.annotation.Coordinate**

`bpsIntersection, compareTo, findLastBase, getChromosome, getStart, getStop, intersects, intersects, parseFile, readBinary, setChromosome, setStart, setStop, splitByChromosome, subtract, toString, writeBinary`

**Methods inherited from class java.lang.Object**

`equals, getClass, hashCode, notify, notifyAll, wait, wait, wait`

**Constructor Detail**

### ScoredCoordinate

```
public ScoredCoordinate(java.lang.String chromosome,
                        int start,
                        int stop,
                        double score)
```


**Method Detail**

### makeRanked

```
public static ScoredCoordinate[] makeRanked(Coordinate[] c)
```

:   Returns an array of ScoredCoordinate where the score is the index position of the Coordinate[].

---


### splitByChromosome

```
public static java.util.HashMap splitByChromosome(ScoredCoordinate[] sortedCoordinates)
```

:   Split by chromosome into a HashMap of chromosome:ScoredCoordinate[].
    Don't forget to sort!

---


### getScore

```
public double getScore()
```

---


### setScore

```
public void setScore(double score)
```


---


|  |  |  |
| --- | --- | --- |
| **Overview** \| **Package** \| **Class** \| **Use** \| **Tree** \| **Deprecated** \| **Index** \| **Help** | |  |
| **PREV CLASS**   **NEXT CLASS** | **FRAMES**    **NO FRAMES**     **All Classes** |  |
| SUMMARY: NESTED \| FIELD \| CONSTR \| METHOD | DETAIL: FIELD \| CONSTR \| METHOD |  |


---
